# Supplementary material for: Assessment of Biological Activity of 28-Homobrassinolide via a Multi-Level Comparative Analysis
Source: Int J Mol Sci. 2023 May 27;24(11):9377. doi: 10.3390/ijms24119377 (PMC10253320; doi:10.3390/ijms24119377)
Supplement: Supplementary file 1 [file ijms-24-09377-s001.zip › Supplemantary materials for IJMS.pptx]

## Slide 1
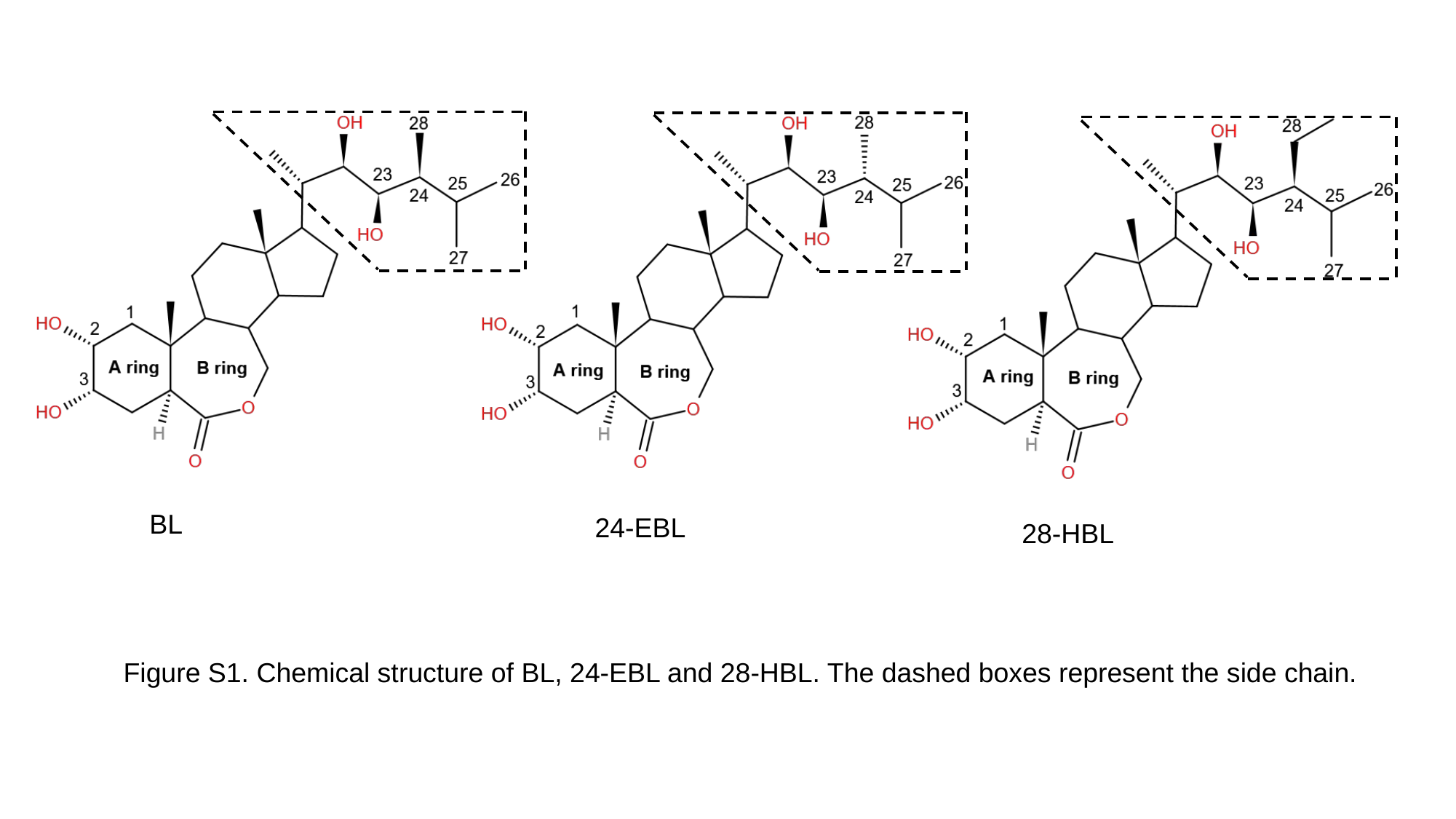

BL
28-HBL
24-EBL
Figure S1. Chemical structure of BL, 24-EBL and 28-HBL. The dashed boxes represent the side chain.

## Slide 2
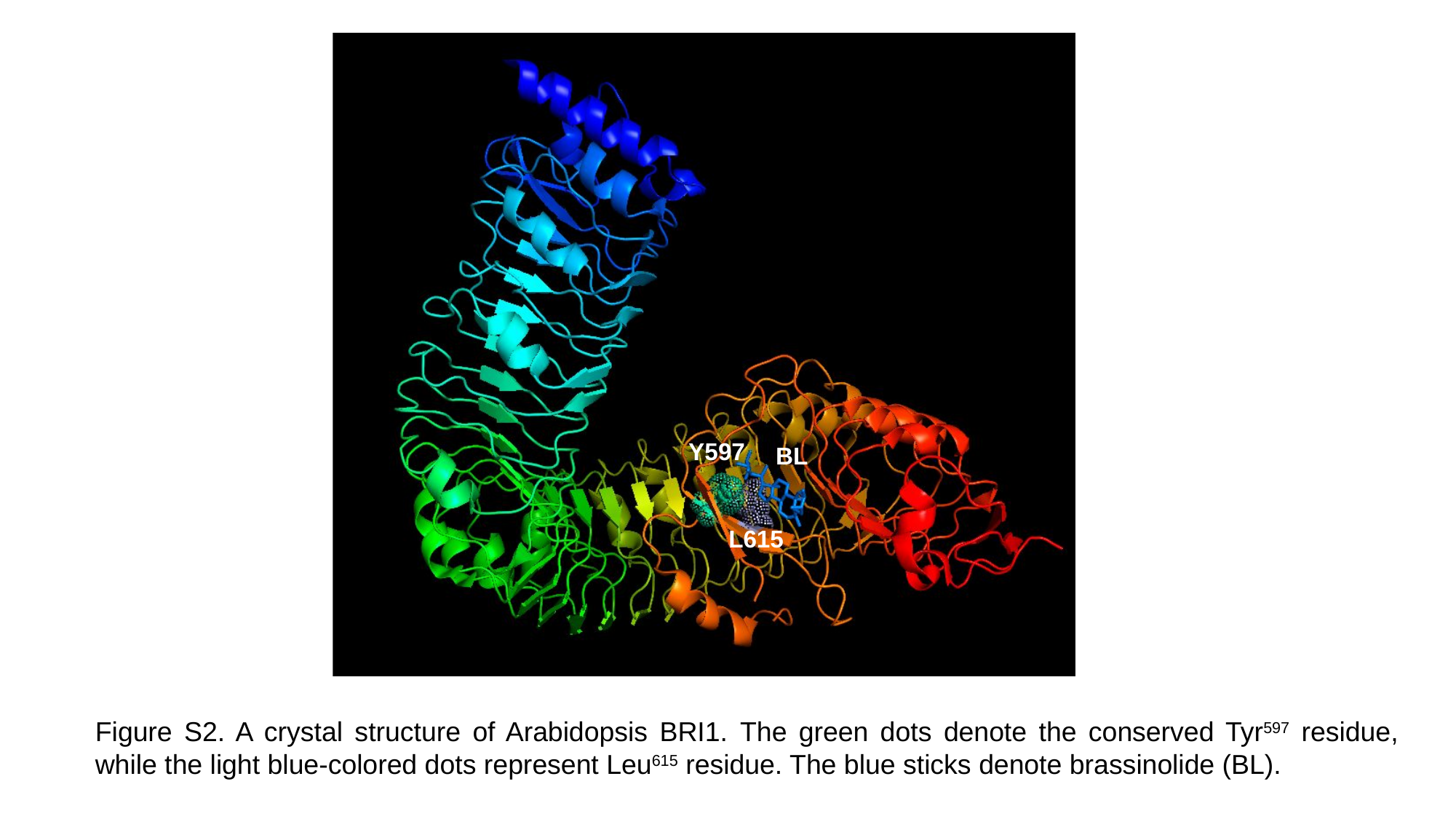

Y597
BL
L615
Figure S2. A crystal structure of Arabidopsis BRI1. The green dots denote the conserved Tyr597 residue, while the light blue-colored dots represent Leu615 residue. The blue sticks denote brassinolide (BL).

## Slide 3
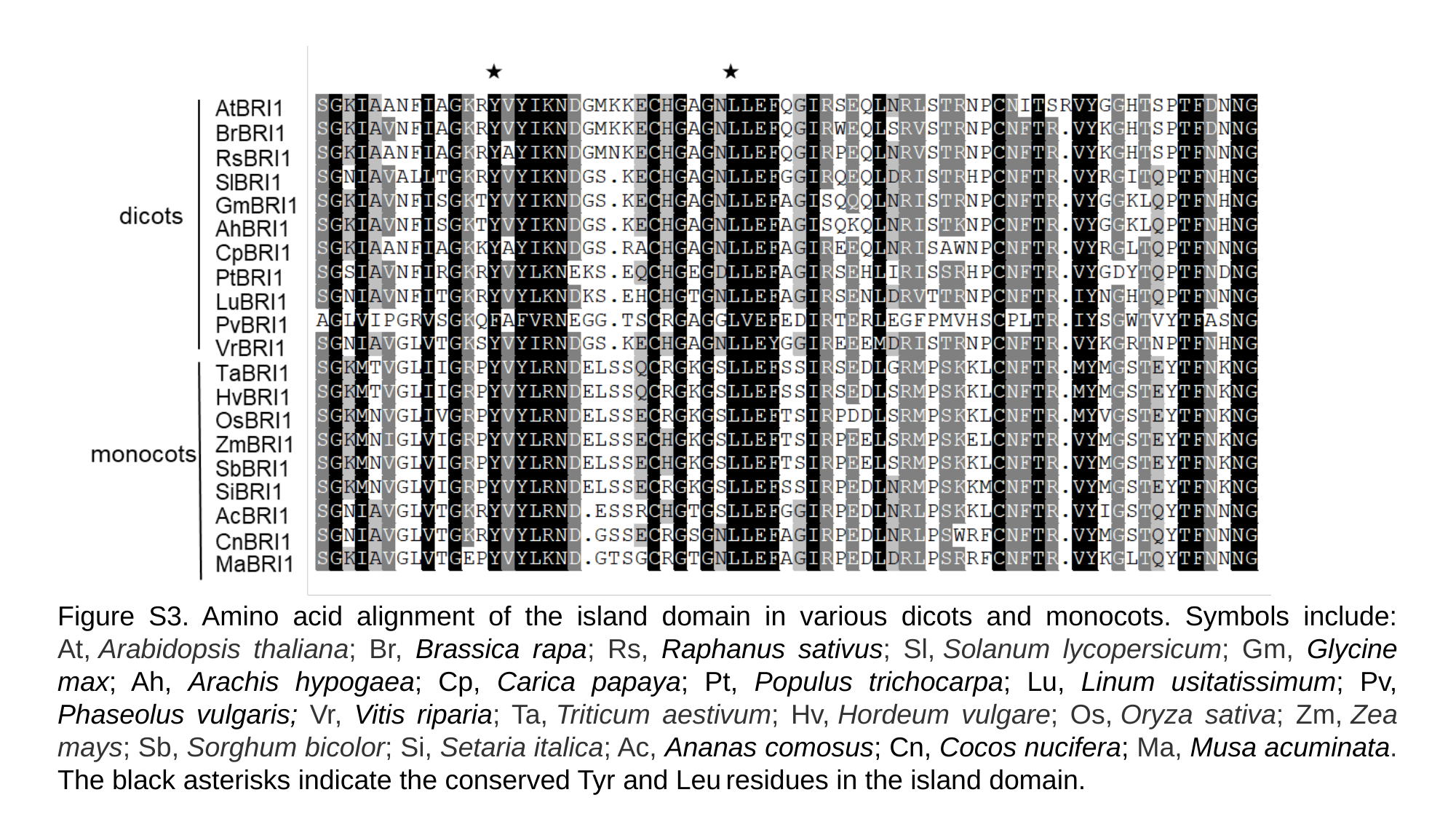

Figure S3. Amino acid alignment of the island domain in various dicots and monocots. Symbols include: At, Arabidopsis thaliana; Br, Brassica rapa; Rs, Raphanus sativus; Sl, Solanum lycopersicum; Gm, Glycine max; Ah, Arachis hypogaea; Cp, Carica papaya; Pt, Populus trichocarpa; Lu, Linum usitatissimum; Pv, Phaseolus vulgaris; Vr, Vitis riparia; Ta, Triticum aestivum; Hv, Hordeum vulgare; Os, Oryza sativa; Zm, Zea mays; Sb, Sorghum bicolor; Si, Setaria italica; Ac, Ananas comosus; Cn, Cocos nucifera; Ma, Musa acuminata. The black asterisks indicate the conserved Tyr and Leu residues in the island domain.

## Slide 4
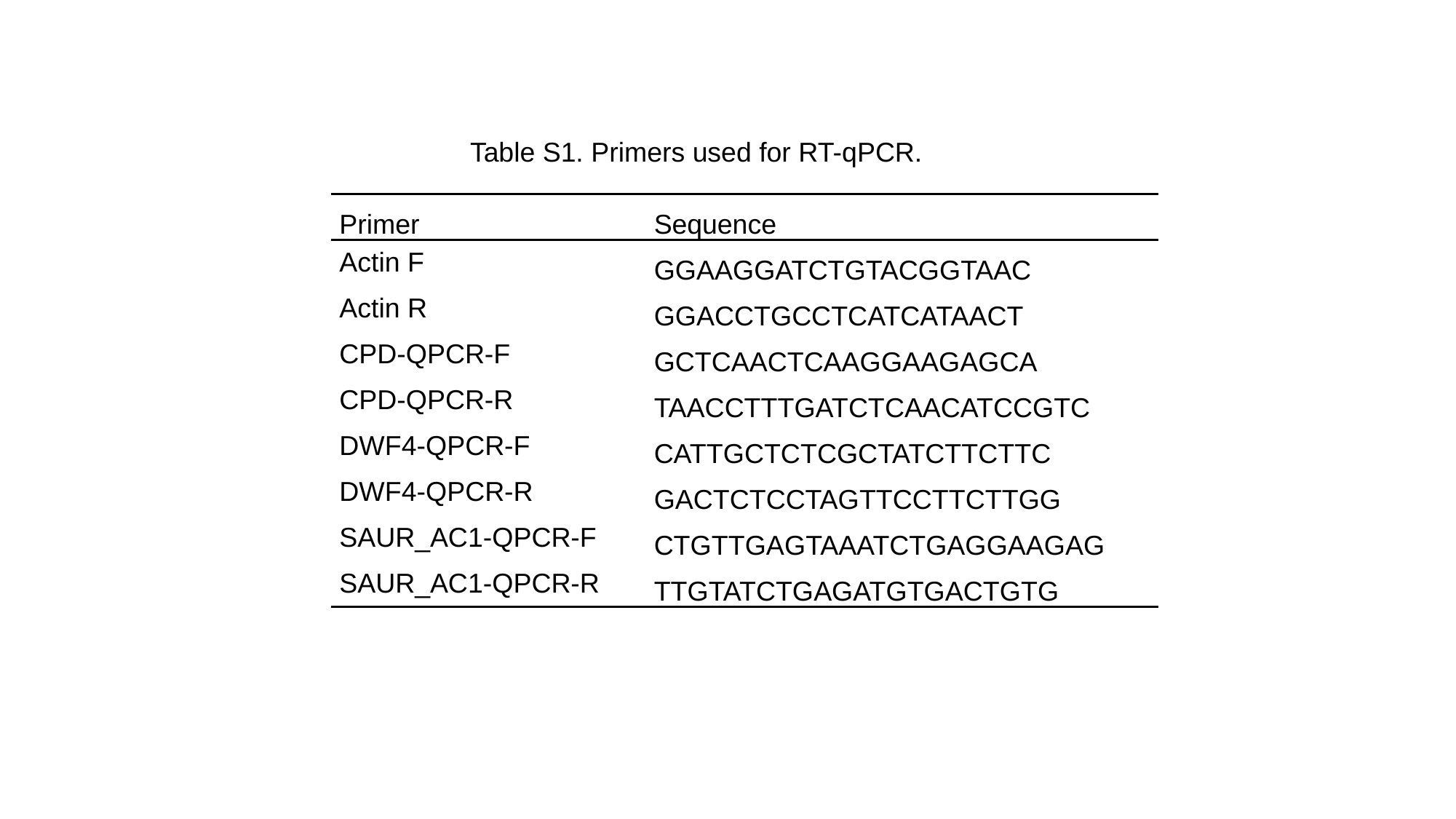

Table S1. Primers used for RT-qPCR.
| Primer | Sequence |
| --- | --- |
| Actin F | GGAAGGATCTGTACGGTAAC |
| Actin R | GGACCTGCCTCATCATAACT |
| CPD-QPCR-F | GCTCAACTCAAGGAAGAGCA |
| CPD-QPCR-R | TAACCTTTGATCTCAACATCCGTC |
| DWF4-QPCR-F | CATTGCTCTCGCTATCTTCTTC |
| DWF4-QPCR-R | GACTCTCCTAGTTCCTTCTTGG |
| SAUR\_AC1-QPCR-F | CTGTTGAGTAAATCTGAGGAAGAG |
| SAUR\_AC1-QPCR-R | TTGTATCTGAGATGTGACTGTG |
